# Food and beverage selection in children’s sports arenas in Norway: a cross-sectional study

**DOI:** 10.1017/S1368980024000818

**Published:** 2024-04-04

**Authors:** Lisa Garnweidner-Holme, Yngvild Frivold, Gigja Max, Kristin Fjæra, Therese Fostervold Mathisen, Mari Charlotte Wik Myhrstad

**Affiliations:** 1 Department of Nursing and Health Promotion, Faculty of Health Sciences, P.O. 4, St. Olavs Plass, Metropolitan University, 0130 Oslo, Norway; 2 Greenudge Health AS, Oslo, Norway; 3 Faculty of Health, Welfare, and Organization, Østfold University College, Fredrikstad, Norway

**Keywords:** Sport arenas, Healthy food, Children, Cross-sectional study, Food environment, Food selection

## Abstract

**Objective:**

To assess the selection of foods and beverages in children’s sports arenas in Norway.

**Design:**

A cross-sectional study design with a digital questionnaire was used. Descriptive statistics were used to present the results. Moreover, Pearson’s *χ* ^2^ tests examined the factors that could aid in distinguishing clubs with healthy or unhealthy consumables.

**Setting:**

Children’s sports clubs in Norway.

**Participants:**

Representatives from 301 children’s sports clubs in Norway answered the questionnaire between September and November 2021.

**Results:**

In total, 89·4% of the participating sports clubs (*n* 301) offered soda drinks with sugar. Most of the sports clubs (88 %) reported to offer batter-based cakes such as pancakes and waffles and 63·8 % offered cakes. Furthermore, 47·5% sold hot dishes with processed meat, such as hamburgers and hot dogs. More than 80% of the sports clubs offered sweets and snacks, while 44·5% did not offer fruits, vegetables and/or berries. Notably, the important factors that distinguished sports clubs with healthier food selections from those with unhealthier selections were the presence of guidelines for the food offered and purchase agreements with food suppliers.

**Conclusions:**

Educational, governmental guidelines for the promotion of healthy eating and establishing agreements with suppliers of healthier foods could help to overcome barriers to unhealthy food selection.

The prevalence of overweight and obesity in children has increased significantly worldwide^(1)^. Recent global estimates suggest that 40 million children under the age of 5 years and more than 330 million children and adolescents aged 5–19 years were overweight or obese in 2016^(2)^. Physical inactivity and/or energy-rich and nutrient-poor diets contribute to rapid weight gain in early childhood and exacerbate the risk factors for chronic disease in children^(3)^. In the short term, children who are overweight or obese are more likely to suffer from psychological co-morbidities, such as depression and behavioural disorders^(4)^. Moreover, being overweight and obese in childhood increases the long-term risk for the development of chronic diseases, such as type 2 diabetes^(5)^. Initiatives to increase physical activity and promote healthy diets are, according to the WHO^(6)^, the most important measures to revise trends in childhood overweight and obesity.

Sports arenas are important avenues for the promotion of healthy behaviours in children and adolescents^(7–9)^. In Norway, participation in organised sports has been associated with a decreased likelihood of unhealthy lifestyle habits among adolescents^(9)^. This points to an optimal opportunity to affect a large number of adolescents, as more than 70 % of Norwegian adolescents participate in children and youth sport^(10)^. However, several international reports state that sports arenas are often used to promote energy-dense and nutrient-poor food and beverages that are not in line with nutrition guidelines^(11–15)^. Other countries, such as Canada and New Zealand, have implemented and evaluated initiatives for guidelines on food offers at the sports arenas^(16,17)^. No national or international guidelines exist in Norway. A scoping review investigating children’s and parents’ opinions of the sport-related food environment found that many children and parents consider the environment neither conducive nor supportive of their children’s healthy food behaviours^(8)^. In New Zealand, parents often experience unhealthy food sponsorship in sports arenas^(18)^. Additionally, club managers and parents participating in a qualitative study in Norway described food selection at the handball halls as unhealthy and wanted a healthier selection^(14)^. Westberg et al. identified the limited availability of healthy options and the presence of unhealthy food and beverage sponsorship as key factors that contribute to unhealthy food choices in sports arenas^(19)^. Importantly, such unhealthy food selections neither promote health nor support sports performance and recovery, as highlighted by current sports nutrition guidelines^(20,21)^.

Even though sports arenas are often characterised by an obesogenic food environment, the systematic investigation of food selection in sports arenas and the exploration of distinguishing factors between clubs with a healthy or unhealthy food selection is lacking. In Norway, several sports clubs share access to a sport arena (i.e. an indoor sport hall including outdoor sporting facilities, often a property of the regional sport federation or the municipality), and each sports club often holds a sales place that are only occasionally opened depending on the size of the club or, most often, the occasion of an event. The sales are often arranged on a volunteering basis, where parents help out with food preparation and sale during opening hours. Such an arrangement allows the profits from the sales place to contribute to the income of sports clubs, making this venture an important source of income^(22)^. Additionally, some clubs have purchase agreements with the food industry. In this study, we assessed food selection in 301 sports clubs in Norway. The aim of this explorative study was to assess the selection of foods and beverages at children’s sports arenas in Norway, which could aid in the better identification of initiatives to facilitate healthier food offers.

## Methods

### Study design, selection and recruitment of participating sports clubs

**Fig. 1:**
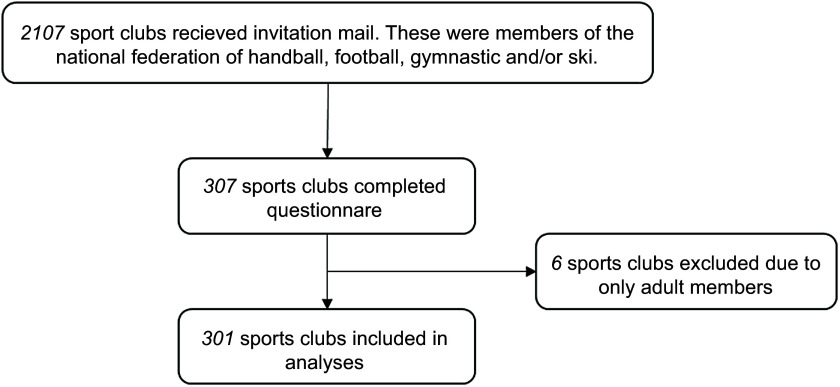
The flow chart of the recruitment process

A cross-sectional study design was used to characterise food and beverage selection in sports arenas for children and adolescents using a digital questionnaire. To reflect the largest organised sports for children and adolescents in Norway and to accommodate different sporting cultures such as team and individual sport and outdoor and indoor sports, sports clubs affiliated with the national sports federations of handball, football, gymnastics and/or skiing were included. Only sports clubs with children and adolescents (6–19 years old) were included. We aimed to include clubs from rural and urban settings from all of the eleven counties in Norway. To contact the persons responsible for the management of the sales places, the researchers at OsloMet got in touch with the national sports federations to reach out to its members. However, only the handball and gymnastic federations responded. The questionnaire was sent by email to all the club members from these sports federations and a total of 814 (handball) and 395 (gymnastics) clubs received the email. To reach the ski clubs, the national ski federation informed its clubs of a link to the questionnaire through their official home page. Additionally, we randomly selected one-third of the cross-country ski clubs from their home page by selecting every third club from the list. A total number of 314 of around 950 clubs were selected and sent email invitations with a link to the questionnaire. The same approach was used to recruit football clubs. Every third and a total of 584 out of 1731 football clubs were randomly selected from the official home page of the football federation, and the invitation was emailed with the link to the questionnaire. Of the total number of sports clubs (2107) that received the email invitation and questionnaire, 307 completed the questionnaire (14·6 %). Six of these clubs were excluded because they had only adult members. We did not sample person-sensitive data in order to reduce the barrier for responding to the survey and to avoid GDPR considerations. The flow chart of the recruitment process is illustrated in Fig. 1.

### Questionnaire

The digital questionnaire was developed using Nettskjema at OsloMet from September 2021 to November 2021. Nettskjema was developed to conduct online surveys and is operated by the University Information Technology Centre (USIT) at the University of Oslo (https://www.uio.no/english/services/it/adm-services/nettskjema/).

The questionnaire was developed to assess the selection of foods and beverages offered in sports arenas. The questions and response-alternatives were developed based on expertise within the research group (most former athletes, most current parents of young athletes, all active researchers within sport and some serving as team/club trainers). The questionnaire was tested for clarity by three participants who were connected to different sports clubs. Their feedback was implemented in the final version of the questionnaire.

The survey included questions related to the general information of the participating sports clubs and information on the food facility and sale environment. The questionnaire comprised two parts. Part 1 included ‘yes’ or ‘no’ questions, such as whether they had a stand for food and beverages associated with their sports club. If answered ‘yes’ to this question, they were automatically forwarded to part 2. Part 2 included four sections with questions and listed-response alternatives, including some comment opportunities related to the background information of the sports clubs (section 1), general information about the sales place (section 2), the selection of foods and beverages sold (section 3) and factors that could impact the selection of food and beverages in the sports arena (section 4). The factors included written guidelines to promote healthy food and beverages at the sports club, purchase agreements with food suppliers, geographical localisation and/or sports club size (measured as the number of members).

In this study, a canteen refers to a sales place with kitchen, whereas a kiosk/café is a smaller sales place offering selected food and beverages. A dispenser is a mobile automate/dispenser selling selected foods and drinks. An informal stand refers to a temporally and mobile sales place, such as a table, that is removed after an event.

To identify factors that could potentially influence the selection of the food and beverages, we selected some products typically offered at the sport arena. These were characterised as healthy or unhealthy options in line with the official nutritional and dietary guidelines^(23)^. The selected healthy options (i.e. low in sugars, fats and salts and/or high in vitamins, minerals, fibres and antioxidants) included bread/rolls/baguettes/crispbread, smoothie, fruits and healthier baked goods (baked with whole meal and/or less added sugar and/or less saturated fat). The unhealthy foods and beverages included soda drinks with sugar (sport drinks and energy drinks were not included), processed meat, such as hamburgers, hot dogs, nuggets and snacks, including chocolate, chips, sweets. A full list of the selected products included in the analyses can be found in supplementary Table 1.

In the questionnaire, the responders were provided explanations on certain food products when appropriate. Briefly, healthier baked products refer to goods baked with whole meal and/or less added sugar and/or less saturated fat. Allergies/religious-specific foods include products categorised as gluten-free, lactose-free or halal prepared products. Batter-baked cakes refer to waffles and cakes. A complete overview of the listed food and beverages is given in the Supplementary Table 1.

### Statistical analyses

Descriptive statistics were produced by Microsoft Office Excel 365 to present the background information of the participating sports clubs, information about their sales places and the selection of food and beverages. These data are summarised and presented as numbers and percentages. Pearson’s *χ* ^2^ test for independence was used to determine the relationships among several factors and the availability of selected healthy/unhealthy options (described above) in the sports arena. IBM SPSS version 27.0 was used for Pearson’s *χ* ^2^ test for independence analyses. *P* < 0·05 was considered statistically significant.

## Results

### Description of the participating sports clubs and their sales places

A total of 301 sports clubs were included in the current study (Fig. 1). The majority (61·1 %) of the sports clubs were members of the handball federation, whereas 45·8 %, 20·9 % and 16·6 % of the sports clubs were members of the football, gymnastics and ski associations, respectively (Table 1). The sports clubs were located in different parts of Norway, representing all eleven Norwegian counties. Most of the sports clubs (40·2 %) had 200–500 members in total (Table 1).

**Table 1:** Description of the participating sports clubs. The results are given in numbers and as % of the total number of sports clubs (*n* (%)

About the sports clubs (*n* 301)	*n*	%
National sport federation*		
Handball	184	61·1 %
Football	138	45·8 %
Gymnastic	63	20·9 %
Ski	50	16·6 %
Other	27	9 %
Geographical localisation (County)		
Agder	13	4·3 %
Innlandet	25	8·3 %
Møre og Romsdal	23	7·6 %
Nordland	14	4·7 %
Oslo	15	5 %
Rogaland	31	10·3 %
Troms and Finnmark	15	5 %
Trøndelag	33	11 %
Vestfold and Telemark	24	8 %
Vestland	30	10 %
Viken	78	25·9 %
Total members		
< 50	3	1 %
50–199	57	18·9 %
200–499	121	40·2 %
500–999	84	27·9 %
1000–2999	34	11·3 %
> 3000	2	0·7 %

* Several of the sports clubs were members of more than one federal sport association.

A detailed description of the sales places, including the type and how they are operated, is presented in Table 2. A total of 86·7 % of sports clubs had a kiosk/cafe, whereas only 14 % had access to a canteen with a kitchen. More than 77 % of the clubs were based on and operated by voluntary work, and only 1·7 % of the sports clubs were reported to be run by private operators. Foods and beverages were usually bought directly from a local grocery store (62·5 %) and/or provided and prepared by the parents of the team members (41·9 %). Additionally, 20·3 % of the sports clubs reported buying products directly from suppliers for specific food concepts, such as fruit and vegetable ((BAMA; a Norwegian company distributing fruit and vegetables) or dairy products (TINE AS; a Norwegian diary company). More than 80 % of the sports clubs reported selling foods and beverages only at special sports events or arrangements, while 10·3 % offered foods and beverages daily (Table 2).

**Table 2:** Description of the kiosks and sales places at the participating sports clubs. The results are given in numbers and as % of the total number of sports clubs (*n* (%))

About the sales place (*n* 301)	*n*	%
What kind of sales place?*		
Kiosk/café†	261	86·7 %
Informal stand (temporally and mobile sales place)	76	25·2 %
Canteen with kitchen	42	14 %
Automat/dispenser (mobile sales place)	23	7·6 %
Who operates/runs the sale place*		
Voluntary people associated to the sports club	234	77·7 %
Sport clubs	134	44·5 %
Municipality	7	2·3 %
Private operators	5	1·7 %
Others (foundation)	1	0·3 %
The food and beverages are bought…*		
Through the local grocery	188	62·5 %
By the parents	126	41·9 %
Through suppliers for specific food concepts (such as fruit and vegetables (BAMA.no) and dairy products (Tine.no)	61	20·3 %
Others	2	0·7 %
When are the sale place opened?*		
At special sports events or arrangements	263	83·4 %
Weekends	41	13·6 %
Every day	31	10·3 %
Other	10	3·3 %

* Multiple options possible.

† A sales place offering selected products depending on facilities.

Most of the sports clubs reported having access to kitchen facilities with a fridge, waffle- and toast iron, oven, freezer, hob, dishwasher and microwave (Table 3).

**Table 3:** Overview of which kitchen facilities at the sales place. The results are given in numbers and as % of the total number of sports clubs (*n* (%))

	*n*	%
Access to kitchen facilities? (*n* 301)		
Yes	257	85·4 %
No	44	14·6 %
What kind of facilities?*		
Fridge	233	77·4 %
Waffle- and toast iron	231	76·4 %
Oven	206	68·4 %
Freezer	205	68·1 %
Hob	203	67·4 %
Dishwasher	202	67·1 %
Microwave	172	57·1 %
Smoothie blender	45	14·9 %
Other	29	9·6 %

* Multiple options possible.

Approximately half of the sports clubs were sponsored with foods and beverages (Table 4). Of these, 38·1 % were sponsored with foods and beverages by the parents of the members and 20·9 % and 12·9 % from private suppliers or groceries, respectively. Furthermore, 39·5 % of the sports clubs reported having purchasing agreements with suppliers for specific food concepts. Most clubs stated that they had income from the sale of food products (Table 4).

**Table 4:** Overview of income and access to free/sponsored foods and beverages. The results are given in numbers and as % of the total number of sports clubs (*n* (%))

	*n*	%
Income from the sale place (given in NOK)		
No income	7	2·3 %
< 10·000	31	10·3 %
10 000–49 000	88	29·2 %
50 000–99 000	67	22·3 %
100 000–249 000	67	22·3 %
250 000–500 000	25	8·3 %
>500 000	16	5·3 %
Access to sponsored products?		
Yes	143	47·5 %
No	158	52·5 %
If Yes, who is the sponsor? (*n* 139)*		
Parents	53	38·1 %
Local groceries	18	12·9 %
Private suppliers	29	20·9 %
Others	44	31·7 %
Agreements with suppliers for food concepts?		
Yes	119	39·5 %
No	182	60·5 %

* Multiple options possible, 100 NOK is approximate 8·7 EUR, 9·4 USD and 7·4 GBP (August 2023).

The sports clubs were also asked whether they had written guidelines regarding the selection of products offered at the sales place (Table 5). Only 18·9 % of the sports clubs reported having such guidelines. These guidelines were related to the purchase and sales (40 %), nutritional content (36 %), food and beverage types (32 %) and recipes (22 %) of the offered products. Approximately 24 % of the sports clubs that reported to have guidelines reported to have guidelines related to more than one of these options (data not shown).

**Table 5:** Overview of sports clubs that have guidelines or written advice to guide the selection of food and beverages at the sale place. The results are given in numbers and as % of the total number of sports clubs (*n* (%))

	*n*	%
Do the sports clubs have guidelines or written advices? (*n* 301)		
Yes	57	18·9 %
No	244	81·1 %
The guidelines/advices were related to (*n* 50)*		
Purchase and sales of products	20	40 %
Nutritional content of products	18	36 %
Type of products offered	16	32 %
Recipes	11	22 %
Other	1	4 %

* Multiple options possible.

### Description of the selection of foods and beverages

**Fig. 2:**
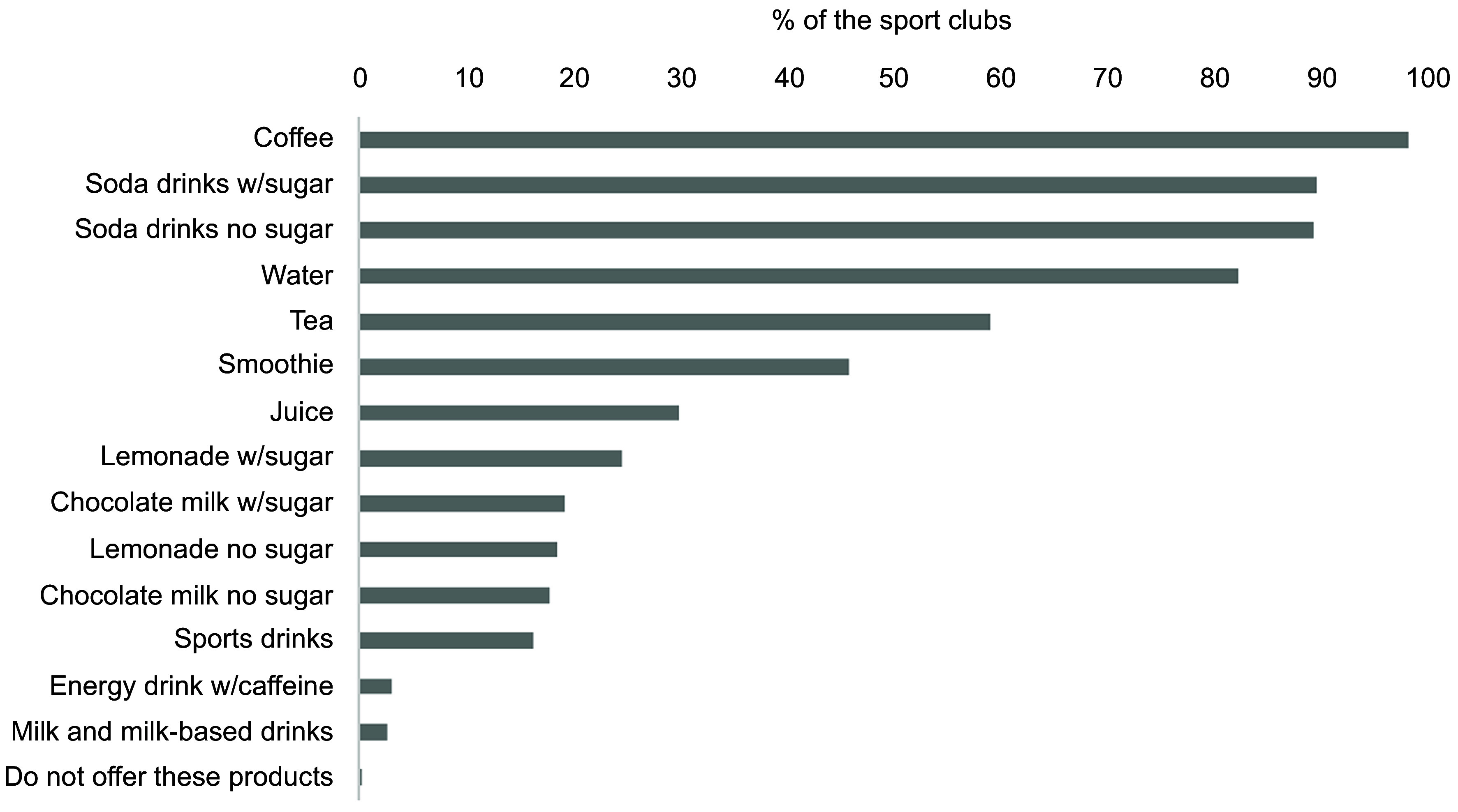
Overview of the selection of beverages offered at the sales place. The results are shown as % of the sports clubs that offer the product out of the total number of sports clubs (*n* 301). Multiple options were possible. w/sugar: with sugar

**Fig. 3:**
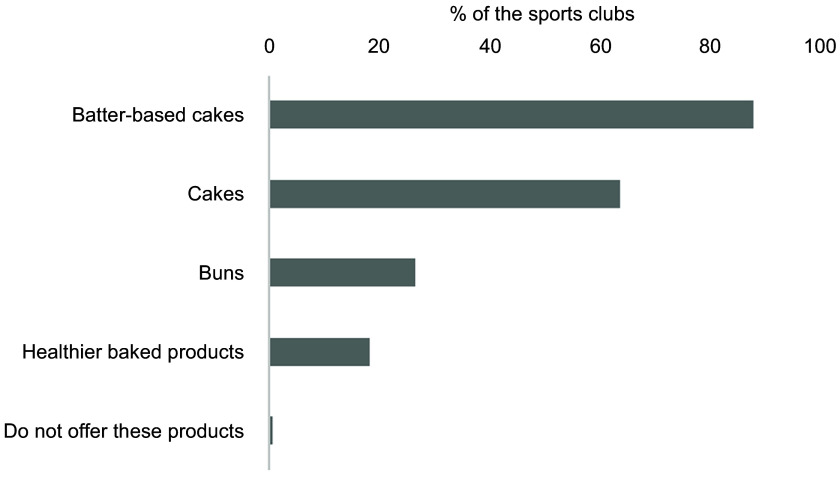
Overview of the selection of sweet cookies and pastries offered at the sales place. The results are shown as % of the sports clubs that offer the product out of the total number of sports clubs (*n* 301). Multiple options were possible. Healthier baked products refer to goods baked with whole meal and/or with less added sugar and/or less saturated fat. Batter-based cakes include waffles and pancakes

**Fig. 4:**
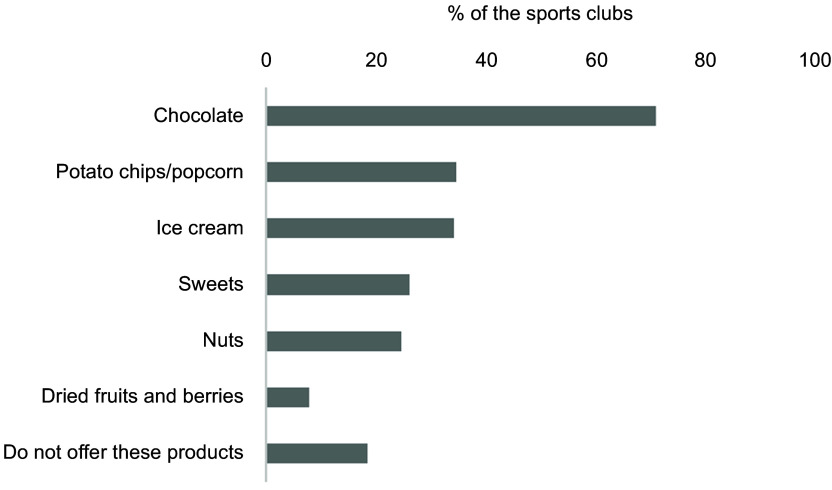
Overview of the selection of snacks offered at the sports arena. The results are shown as % of the sports clubs that offer the product out of the total number of sports clubs (*n* 301). Multiple options were possible

**Fig. 5:**
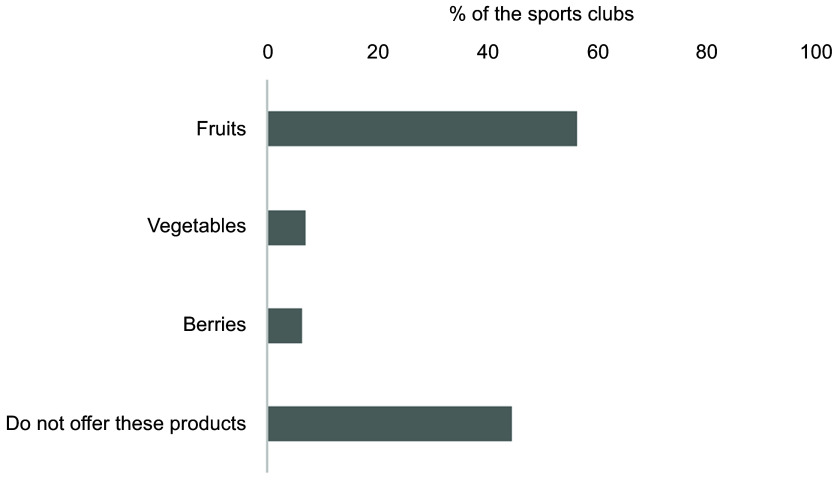
Overview of the number of sports clubs offering fruit, vegetables and/or berries out of the total number of sports clubs (*n* 301). Multiple options were possible

**Fig. 6:**
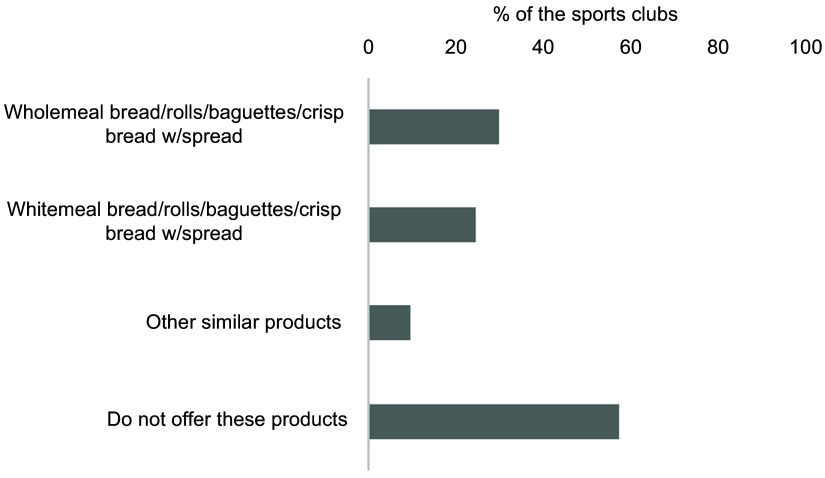
Overview of the selection of bread, rolls, baguettes and crispbread with spreads. The results are shown as % of the sports clubs that offer the product out of the total number of sports clubs (*n* 301). Multiple options were possible. Other similar products refer to products not listed to give the responders the possibility to add products. Several of the responders added toast

**Fig. 7:**
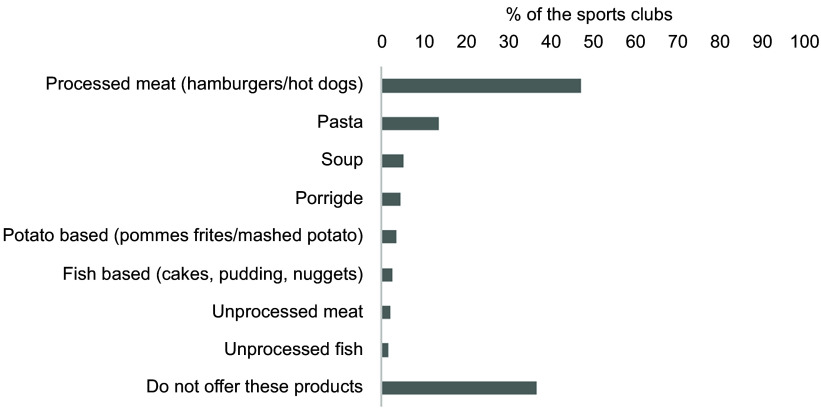
Overview of the selection of hot dishes. The results are shown as % of the sports clubs that offer the product out of the total number of sports clubs (*n* 301). Multiple options were possible

**Fig. 8:**
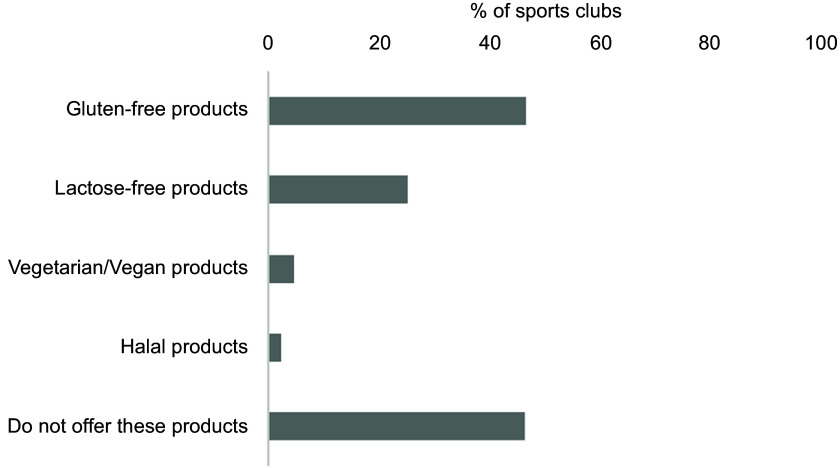
Overview of food products taking food allergies and intolerances and religion into account. The results are shown as % of the sports clubs that offer the product out of the total number of sports clubs (*n* 301). Multiple options were possible

An overview of the selection of foods and beverages offered in the sports arenas is presented in Figs 2–8. Coffee and soda drinks with and without sugar and water were offered by most sports clubs (< 80 %) (Fig. 2). Concurrently, < 50 % of the sports clubs offered smoothies, juices, lemonades, sports drinks and energy drinks. Milk and milk-based drinks, except for chocolate milk, were offered in < 3 % of the included sports clubs.

An overview of the selection of sweet cookies and pastries offered in the sports arena is presented in Fig. 3. Most sports clubs reported selling these products, with batter-based cakes (e.g. waffles and pancakes) and cakes sold by 88 % and 63·8 % of the clubs, respectively. Only 18. 3 % of the sports clubs reported offering a healthier alternative to these products, such as products baked with whole meal and/or less added sugar and/or less saturated fat (Fig. 3).

Furthermore, most sports clubs offered sweets and snacks. Chocolate, potato chips and popcorn, ice cream and sweets were offered by 71·1 %, 34·6 %, 34·2 % and 26·2 %, respectively. However, nuts and dried fruits/berries were sold by only 24·6 % and 8 % of the reporting sports clubs, respectively (Fig. 4).

Fruits, vegetables and berries were offered by 56·5 %, 7 % and 6·3 % of the sports clubs, with approximately half of them not offering any of these options (Fig. 5).

Figure 6 presents the selection of bread, rolls, baguettes and crispbread with spreads, and 57·5 % of the sports clubs included did not offer these products. Wholemeal bread, rolls, baguettes and crispbread with spread were offered by 29·9 % of the sports clubs, whereas 24·6 % of the clubs reported selling these products made of white flour.

Of the included sports clubs, 47·5 % reported selling hot dishes with processed meat, such as hamburgers, hot dogs and nuggets. Dishes with pasta and soups were offered by 13·6 % and 5·3 %, respectively. However, 36·9 % of the sports clubs did not offer hot dishes, and < 3 % offered unprocessed meat and fish products (Fig. 7).

Figure 8 shows that 46·5 % of the sports clubs did not offer food products that took food allergies and intolerances and religion into account. Gluten- and lactose-free food products were offered by 46·8 % and 25·2 % of sports clubs, respectively. Furthermore, only 4·7 % and 2·3 % reported selling vegetarian/vegan and halal food products, respectively.

### Factors that could impact the availability of healthy/unhealthy food and beverage selection

The analysis results revealed that having guidelines for the selection of food and beverages and purchasing agreements with suppliers of food concepts positively influenced the selection of food and beverages as a healthier option (Tables 6–8).

**Table 6:** The relation between written guidelines and the offering of healthy/unhealthy food and drink products

	Total (*n* 301)		Guidelines (*n* 57)		No guidelines (*n* 244)		*P* value
	*n*	%	*n*	%	*n*	%	
Bread/rolls/baguettes/cripsbread*							<0·001
Yes	128	42·5	37	64·9	91	37·3	
No	173	57·5	20	35·1	153	62·7	
Smoothie							<0·001
Yes	138	45·8	40	70·2	98	40·2	
No	163	54·2	17	29·8	146	59·8	
Healthier baked goods							0·007
Yes	55	18·3	18	31·6	37	15·2	
No	246	81·7	39	68·4	207	84·8	
Fruits							<0·001
Yes	171	56·8	46	80·7	125	51·2	
No	130	43·2	11	19·3	119	48·8	
Processed meat							0·110
Yes	143	47·5	33	57·9	110	45·1	
No	158	52·8	24	42·1	134	54·9	
Soda drinks with sugar							0·244
Yes	269	89·4	48	84·2	221	90·6	
No	32	10·6	9	15·8	23	9·4	
Snacks†							0·676
Yes	245	81·4	48	84·2	197	80·7	
No	56	18·6	9	15·8	47	19·3	
Allergy/intolerances/religion							0·018
Yes	161	53·5	39	68·4	122	50	
No	140	46·5	18	31·6	122	50	

* Includes bread/rolls/baguettes/crispbread with and without spread.

† Includes chocolate, potato chips and popcorn, ice-cream, sweets, nuts, dried fruits and berries. Healthier baked goods are baked with whole meal and/or less added sugar and/or less saturated fat.

**Table 7:** The relation between agreement with supplier for food concepts and the offering of healthy/unhealthy food products

	Total (*n* 301)		Agreement (*n* 119)		No agreement (*n* 182)		*P* value
	*n*	%	*n*	%	*n*	%	
Bread/rolls/baguettes/crispbread*							0·018
Yes	128	42·5	61	51·3	67	36·8	
No	173	57·5	58	48·7	115	53·2	
Smoothie							<0·001
Yes	138	45·8	74	62·2	64	35·2	
No	163	54·2	45	37·8	118	64·8	
Healthier baked goods							0·018
Yes	55	18·3	30	25·2	25	13·7	
No	246	81·7	89	74·8	157	86·3	
Fruits							0·034
Yes	171	56·8	77	64·7	94	51·6	
No	130	43·2	42	35·3	88	48·4	
Processed meat							0·474
Yes	143	47·5	53	44·5	90	49·5	
No	158	52·8	66	55·5	92	50·5	
Soda drinks with sugar							0·745
Yes	269	89·4	105	88·2	164	90·1	
No	32	10·6	14	11·8	18	9·9	
Snacks†							0·021
Yes	245	81·4	105	88·2	140	76·9	
No	56	18·6	14	11·8	42	23·1	
Allergy/intolerances/religion							0·252
Yes	161	53·5	69	58·0	92	50·5	
No	140	46·5	50	42·0	90	49·5	

* Includes bread/rolls/baguettes/crispbread with and without spread.

† Includes chocolate, potato chips and popcorn, ice-cream, sweets, nuts, dried fruits and berries. Healthier baked goods are baked with whole meal and/or with less added sugar and/or less saturated fat.

**Table 8:** Geographical localisation in Norway

	Whole country (*n* 301)		South (*n* 37)		West (*n* 84)		South East (*n* 118)		Middle (*n* 33)		North (*n* 29)		*P* value
	*n*	%	*n*	%	*n*	%	*n*	%	*n*	%	*n*	%	
Guidelines													0·739
Yes	57	18·9	8	21·6	14	16·7	21	17·8	6	18·2	8	27·6	
No	244	81·1	29	78·4	70	83·3	97	82·2	27	81·8	21	72·4	
Agreement with concept													0·023
Yes	119	39·5	15	40·5	33	39·3	57	48·3	7	21·2	7	24·1	
No	182	60·5	22	59·5	51	60·7	61	51·7	26	78·8	22	75·9	

More sports clubs having guidelines offered healthy products such as bread, rolls, baguettes and crispbread with spreads (*P* < 0·001), smoothies (*P* < 0·001), fruits (*P* < 0·001) and healthier alternatives to baked goods (cookies and pastries baked with whole meal and/or less added sugar and/or less saturated fat) (*P* = 0·007) compared with those without the guidelines. Additionally, the sports clubs that had guidelines more often offered products that accounted for food allergies, food intolerances and religion compared with those without guidelines (*P* = 0·018) (Table 6).

Furthermore, sports clubs with supplier agreements offered bread, rolls, baguettes and crispbread with spreads (*P* = 0·018), smoothie (*P* < 0·001), fruit (*P* = 0·034) and a healthier alternative to baked goods (*P* = 0·018), in addition to snacks, such as chocolate, chips and sweets (*P* = 0·021), compared with those without such agreements. (Table 7).

The geographical localisation of the sports clubs that reported to have guidelines did not differ significantly from the sports clubs that did not have guidelines. However, sports clubs that reported having agreements with food suppliers were majorly located in the southern part of Norway (*P* = 0·023) (Table 8).

Analyses related to size of the club did not show any relationship with the availability of the selected healthy foods and beverages (data not shown).

## Discussion

Overall, most participating sports clubs (> 60 %) offered unhealthy food and beverages, which are characterised by sugary drinks, sweets, snacks, baked goods and batter-based cakes. Additionally, 44·5 % of the sports clubs did not offer fruits, vegetables and/or berries in the sports arena. Foods relevant to sports performance and recovery (i.e. sports-related food products and milk products) were less typically offered in these arenas. The important factors that distinguished sports clubs with healthier food options compared with those with less healthy food offerings were the existence of guidelines for the food offered and the presence of purchase agreements with food suppliers. While geographical location did not significantly differ among sports clubs with or without guidelines, it differed among those with purchase agreements and those without.

There is limited knowledge of food selection in children’s sports arenas worldwide^(11)^. In New Zealand, sugar-sweetened beverages have been found to be widely available and promoted among sport-playing children. Moreover, solutions to improve the situation widely include promoting water as the beverage of choice in sports and implementing healthy eating and drinking campaigns in sports clubs^(24)^. Our findings indicate that the potential to use the sports arena for health promotion is not sufficiently met in participating sports clubs. Although more than 80 % of the sports clubs reported selling foods and beverages only at special sports events or arrangements (*v*. the 10·3 % on a daily basis), food selection in the sports arena might influence children’s and their families’ dietary habits^(25)^.

In our survey, most sales places operated only on special occasions and did not cater daily to their customers (i.e. sporting children and adolescents and their parents and supporters). As such, one may argue that the sales place do not have a substantial impact on the health of their customers. However, globally, sport is a very powerful avenue, creating identity, unity and community, that creates a wide impact through the values and messages it promotes. Hence, sponsorship by brands promoting unhealthy foods is problematic in sports^(12,16,18,19,26)^. Cultural changes, professional guidelines implemented widely in the club, preferably by the national federation and support from elite athletes who promote healthy food ideals could form important strategies to enforce a healthy food identity in sports^(16,18,19)^. The impact of actions by athletes was effectively demonstrated by the soccer player Cristiano Ronaldo in one press conference wherein he replaced Coca-Cola with water, causing a major drop in the market value of Coca-Cola and inciting massive press coverage^(27)^.

The WHO has identified a healthy diet as one of the cornerstones to reduce the burden of non-communicable diseases^(28)^, outlining the importance to implement political and structural measures for health promotion^(25)^. Our survey observed a high frequency of sports clubs offering unhealthy food options, underscoring previous sporadic descriptions of the sports arena^(19)^. Thus, it is important to identify strategies and implement government regulations that may facilitate healthier food selections. A previous study found that voluntary nutritional guidelines significantly improved the food environment at recreation and sport facilities^(29)^. However, guidelines may have little impact if the foods are offered by commercial providers in the sports arena (like vending machines), wherein brand agreements and profits run the premises^(7)^. In Norway, clubs often run sales places based on volunteer efforts, thus, the interests of the volunteers (as they are also the customers) have a stronger impact on food selection. By providing information and guidelines, the preferences of the customers may be changed to a favourable and healthier direction. Such attitudinal effects from presenting information have previously been demonstrated^(18,26)^, pointing to a potential favourable change in behaviour if information is concurrently matched with the actual food selection. Participants within a club reported that governmental guidelines would make it easier to change food selection in sports clubs^(14)^. Hence, governmental guidelines might indeed have an impact on a healthier food selection at the sports clubs.

Most of the clubs in the present study stated that major income was generated from the sale of food products, and previous findings have highlighted that the typical selection of unhealthy food was viewed as necessary, as it was preferred by customers^(14)^. However, information and message promotion regarding a healthy diet have also been speculated to change the attitudes of customers^(18,26)^. Furthermore, the presence of information and implementation of guidelines could aid in identifying solutions for food preparation and sale that do not lead to increased food waste or income loss. Other reported reasons for the difficulty in offering healthy food selections are limited facilities and insufficient time to prepare these foods^(14)^. Contrastingly, the current study reported that most sports clubs have access to kitchen facilities with a fridge, waffle and toast iron, oven, freezer, hob, dishwasher and microwave. Hence, user-friendly educational materials about nutrition can inform parents and guardians about the types of food that are appropriate for sports settings and how to prepare healthier alternatives, thereby promoting a healthier food selection^(30)^.

In addition, 47·5 % of the sports clubs were provided with sponsored foods and beverages. The children’s parents, along with local suppliers and groceries, were the sponsors. Unfortunately, we do not have information about the foods and beverages that were sponsored from the local suppliers and groceries. However, sponsorship of unhealthy food products in the sports arena has previously been demonstrated to be a major barrier to healthy food selection in sports arenas^(12,26,31)^. In contrast, this survey finds that clubs offer healthier food selections if they have purchasing agreements with suppliers offering food concepts, such as fruit (BAMA; a Norwegian company distributing fruit and vegetables) and/or dairy products (TINE AS; a Norwegian diary company). Thus, regulations for sponsorships that promote healthy food selection in the sports arena could be an important opportunity for health promotion.

Despite the increased knowledge about performance nutrition and healthy diets in many industrial countries, food selection in sports arenas does not cater to these requirements. This survey and previous findings from other countries^(11,15,29)^ highlight that the obvious opportunities for healthy food selections in the sports arena are by establishing professional guidelines, including nutritional arguments, recipes and cooking advice, and arranging food supply agreements. Thus, governmental efforts must be implemented to enforce cultural changes by engaging the club at the top level (including the federations) and involving communication by elite athletes.

### Study limitations

To our knowledge, this study is the first systematic exploration of food and beverage selection in children’s sports arenas. The study was performed with a non-validated questionnaire owing to the unavailability of a validated questionnaire. It has to be acknowledged that the food selection and background information were self-reported. Nevertheless, the questionnaire was piloted by the research group and a small group of invited sports clubs, who tested the questions for clarity. Explanations for some food and beverages, such as healthier baked goods, were given in the questionnaire to get a more valid result. We used a questionnaire with defined response alternatives and cannot rule out the possibility that we have not been able to map all the products offered. In the results presented, we took the opportunity to identify factors that could potentially promote the selection of food and beverages in a healthier direction. Certain selected items were therefore characterised as healthy and unhealthy in line with the Norwegian food-based guidelines. We were, however, not able to determine the proportion of the healthy and unhealthy goods offered, the sales numbers of these products or the exact nutritional quality of the selected products. The questionnaire only included a question if the clubs had guidelines for the food selection at the sport arena (yes/no). Hence, we do not have any more specific information about what kind of guidelines they had. Furthermore, these analyses do not offer any causal relationship and we cannot rule out the possibility that other factors not analysed could impact the availability of healthy and unhealthy products. Although this study was distributed among most Norwegian sports clubs for children, the results from the 301 clubs included cannot be generalised nationwide. We succeeded to recruit sport arenas across Norway. However, we do not know whether both rural and urban located sports clubs were represented. Furthermore, we did not collect data on the clubs’ economy or socio-economic status of their members to analyse possible factors related to resources on the food selection. Furthermore, data were collected during the COVID-19 pandemic, and restrictions might have influenced the food selection at the participating sports clubs. However, the participants were asked to report their usual food selection.

### Conclusions

This study revealed that a majority of the sports clubs offered foods and beverages that were characterised by sugar-rich drinks, sweetened bakery products and processed meat products, such as hamburgers and hot dogs. Therefore, educational guidelines on how to prepare and offer healthy food in addition to purchasing agreements with suppliers offering healthy food concepts could improve food and beverage selection in children’s sports arenas.

## Supporting information

Garnweidner-Holme et al. supplementary material Garnweidner-Holme et al. supplementary material

## Supplementary material

For supplementary material accompanying this paper visit https://doi.org/10.1017/S1368980024000818
