# Supplementary material for: Food and beverage selection in children’s sports arenas in Norway: a cross-sectional study
Source: Public Health Nutr. 2024 Apr 4;27(1):e115. doi: 10.1017/S1368980024000818 (PMC11036441; doi:10.1017/S1368980024000818)
Supplement: Garnweidner-Holme et al. supplementary material [file S1368980024000818sup001.docx]

**Supplementary Table 1: Overview of categories and listed product in questionnaire.**

| **Category** | **Listed product** | **Information of product** |
| --- | --- | --- |
| ***Beverages*** |  |  |
|  | Coffee | All types of coffee |
|  | Soda drinks w/sugar # |  |
|  | Soda drinks no sugar |  |
|  | Water |  |
|  | Tea |  |
|  | Smoothie * |  |
|  | Juice |  |
|  | Lemonade w/sugar |  |
|  | Chocolate milk w/sugar |  |
|  | Lemonade no sugar |  |
|  | Chocolate milk no sugar |  |
|  | Sports drinks |  |
|  | Energy drink w/caffeine |  |
|  | Milk and milk-based drinks | Whole-, skimmed-, flavored- and soured milk |
| ***Sweet cookies and pastries*** |  |  |
|  | Batter-based cakes | Waffles, pancakes |
|  | Cakes | All types of cakes included cookies |
|  | Buns |  |
|  | Healthier baked goods * | Baked with whole meal and/or with less added sugar and/or less saturated fat |
| ***Snacks*** |  |  |
|  | Chocolate # |  |
|  | Potato chips/popcorn # |  |
|  | Ice cream # |  |
|  | Sweets # |  |
|  | Nuts # |  |
|  | Dried fruits and berries # |  |
| ***Fruits, vegetables, and berries*** |  |  |
|  | Fruits * | Includes whole and chopped variants |
|  | Vegetables | Includes whole and chopped variants |
|  | Berries | Includes whole and chopped variants |
| ***Bread, rolls, baguettes, and crispbread*** |  |  |
|  | Wholemeal * | With spread |
|  | Whitemeal | With spread |
|  | Similar products * | Similar products not on the list (e.g toast) |
| ***Hot dishes*** |  |  |
|  | Processed meat # | Hamburgers, hot dogs, nuggets |
|  | Pasta |  |
|  | Soup |  |
|  | Porridge |  |
|  | Potato based products | Pommes frites and mashed potato |
|  | Fish based products | Fish cakes, fish pudding and fish nuggets |
|  | Unprocessed meat |  |
|  | Unprocessed fish |  |
| ***Allergies/intolerances and religion*** |  |  |
|  | Gluten-free products | Specially marked as gluten-free |
|  | Lactose-free products | Specially marked as lactose-free |
|  | Vegetarian/Vegan products |  |
|  | Halal products |  |

*Considered healthy products and subjected to Pearson Chi-square analyses. # Considered unhealthy products and subjected to Pearson Chi-square analyses.
